# A comprehensive literature scoping review of infection prevention and control methods for viral-mediated gene therapies

**DOI:** 10.1017/ash.2024.1

**Published:** 2024-01-31

**Authors:** Jill E. Blind, Sumit Ghosh, Taylor D. Niese, Julia C. Gardner, Stephanie Stack-Simone, Abigail Dean, Matthew Washam

**Affiliations:** 1 Department of Pharmacy, Nationwide Children’s Hospital, Columbus, OH, USA; 2 Department of Research Safety, Abigail Wexner Research Institute at Nationwide Children’s Hospital, Columbus, OH, USA; 3 Department of Pharmacy, Cincinnati Children’s Hospital Medical Center, Cincinnati, OH, USA; 4 Center for Clinical Excellence, Department of Epidemiology, Nationwide Children’s Hospital, Columbus, OH, USA

## Abstract

**Objective:**

This comprehensive literature scoping review outlines available infection prevention and control (IPC) methods for viral-mediated gene therapies and provides one IPC strategy for the healthcare setting based on a single-center recommendation.

**Methods:**

A team of experts in pharmacy, healthcare epidemiology, and biosafety with experience in viral-mediated gene therapy was assembled within a pediatric hospital to conduct a comprehensive literature scoping review. The comprehensive review included abstracts and full-text articles published since 2009 and utilized prespecified search terms of the five viral vectors of interest: adenovirus (AV), retrovirus (RV), adeno-associated virus (AAV), lentivirus (LV), and herpes simplex virus (HSV). Case reports, randomized controlled trials, and bench research studies were all included, while systematic reviews were excluded.

**Results:**

A total of 4473 case reports, randomized control trials, and benchtop research studies were identified using the defined search criteria. Chlorine compounds were found to inactivate AAV and AV, while alcohol-based disinfectants were ineffective. There was a relative paucity of studies investigating surface-based disinfection for HSV, however, alcohol-based disinfectants were effective in one study. Ultraviolent irradiation was also found to inactivate HSV in numerous studies. No studies investigated disinfection for LV and RV vectors.

**Conclusions:**

The need to define IPC methods is high due to the rapid emergence of viral-mediated gene therapies to treat rare diseases, but published clinical guidance remains scarce. In the absence of these data, our center recommends a 1:10 sodium hypochlorite solution in clinical and academic environments to ensure complete germicidal activity of viral-mediated gene therapies.

## Introduction

Over the last 10 years, novel biological therapies have rapidly emerged as revolutionary treatment options for rare diseases.^1,2^ According to the American Society for Gene and Cell Therapy, over 3,900 gene, cell, and RNA therapies are currently in development across the globe.^3,4^ Within the gene therapy landscape, many of the in vivo genetically modified therapies are formulated with a viral-mediated backbone. Viral vector-mediated gene therapies use modified viruses as drug-delivery vehicles to introduce specific DNA sequences, regulatory RNAs, or other therapeutic substrates into cells^5,6^ Commercial gene therapy agents, such as onasemnogene abeparvovec and voretigene neparvovec, are comprised of an adeno-associated virus (AAV) vector, whereas the oncolytic virus, talimogene laherparepvec, is comprised of a modified herpes simplex virus.^7^ Nearly all gene therapies commercially available use one of three vector types: AAV, adenovirus (AV), or lentivirus (LV).^4,8^ Adeno-associated virus and AV vectors are typically used in gene therapies directly administered to patients by infusion or local administration, with AAV being the most popular vector for areas outside of oncology and vaccines.^4,8^ Lentivirus vectors are typically used for ex vivo therapies, in which cells harvested from a patient are modified in the lab before transplantation.^4,8^

Although these viral-mediated gene therapies are genetically modified to not cause human disease, they all possess the unique property of being biologically active and carry potential biohazardous risks to the healthcare workers who handle them directly—a characteristic not typically seen with traditional pharmaceutical drug formulations.^9,10^ In addition, viral shedding post-infusion may present the possibility of viral transmission to healthcare workers caring for these patients. While the viability for long-term gene expression and the adverse effects of these drugs within their respective patient populations will continue to be monitored in late-phase clinical trials and through post-marketing surveillance, occupational safety data will lag. Further, the limited shedding data reported in early clinical trials and the lack of regulatory guidance describing infection prevention and control (IPC) methods for these therapies ultimately prevent biosafety and healthcare epidemiology professionals from clearly defining post-infusion infection control standards.^1^

With many novel biologic therapies being pushed through fast-track approval pathways, health systems will consequently be challenged to develop on-demand IPC guidance, using limited knowledge and occupational safety data to match both the unique viral vector systems and the quick pace of gene and cell therapy development.^3^ Contact times for kill rates on commercial disinfecting agents could be utilized to provide baseline guidance for defining IPC practices within the institutional setting; however, emerging therapies utilize novel viral vector systems or genetically modified organisms that are not found in the environment and, therefore, do not have corresponding published kill rate data. The current literature scoping review provides a comprehensive analysis of available IPC methods reported for viral-mediated therapeutics. Additionally, we provide one possible strategy for the development of best-practice IPC recommendations for the healthcare setting based on our extensive, single-center experience working in a large pediatric hospital with an associated research institute.

## Methods

A team of pharmacy, healthcare epidemiology, and biosafety experts was identified within the institution and assembled to initiate the project. The viral vectors chosen for the comprehensive literature scoping review included AV, retrovirus (RV), AAV, LV, and herpes simplex virus (HSV). These 5 viruses account for over 56% of all vector systems utilized in clinical trials.^3^ In addition, modified versions of 3 of these viruses are found in commercially approved drugs within the United States (AV, AAV, HSV); the remaining 2 (RV, LV) are frequently utilized for genetic modifications in cellular-based gene therapy but continue to be researched in a variety of clinical applications.^6,11–14^ Three bibliographic databases were chosen for review: Cumulative Index to Nursing and Allied Health Literature; MEDLINE from the National Library of Medicine; and PubMed from the National Library of Medicine. The Laboratory-Acquired Infections (LAI) database from the American Biological Safety Association was also used to acquire research case reports of occupational or environmental infections in research.

Search terms were identified using medical subject headings from the National Library of Medicine. To obtain articles on infection control, the following search terms were defined by the team: disinfect; environmental exposure; occupational exposure; biosafety; infection control; and inactivation. Viral vector search terms were expanded to include various alliterations and included: adenoviridae; adenoviridae vector; AV; AV vector; retroviridae; retroviridae vector; RV; RV vector; adeno-associated virus vector; AAV vector; LV; LV vector; simplex virus; and simplex virus vector. Each viral vector term was individually paired with each infection control term, requiring 70 different search combinations to complete the literature scoping review within the bibliographic databases (Table 1). Viral vector terms alone were utilized to search the LAI database. Search filters were applied to limit results to those published since 2009 and published in English. Both abstracts and full articles were permitted for inclusion.

**Table 1.** Search strategy for comprehensive literature review^a^ ^,^ ^b^

Viral vector of interest	Vector term	Cleaning term
Adenovirus (AV)	Adenovirus vector	Disinfect
	Adenoviridae vector	Environmental exposure
	Adenovirus	Occupational exposure
	Adenoviridae	Infection control
		Inactivation
Retrovirus (RV)	Retrovirus vector	Disinfect
	Retroviridae vector	Environmental exposure
	Retrovirus	Occupational exposure
	Retroviridae	Infection control
		Inactivation
Adeno-associated virus (AAV)	Adeno-associated virus vector OR AAV vector	Disinfect
	Adeno-associated virus OR AAV	Environmental exposure
		Occupational exposure
		Infection control
		Inactivation
Lentivirus (LV)	Lentivirus vector	Disinfection
	Lentivirus	Environmental exposure
		Occupational exposure
		Infection control
		Virus inactivation
Herpes simplex virus (HSV)	Simplex virus vector	Disinfection
	Simplex virus	Environmental exposure
		Occupational exposure
		Infection control
		Virus inactivation

a *Search method for Cumulative Index to Nursing and Allied Health Literature (CINAHL), MEDLINE from the National Library of Medicine, and PubMed from the National Library of Medicine included “[Vector Term] AND [Cleaning Term].”*

b *Search method for Laboratory-Acquired Infections database included “[Vector Term].”*

Case reports, randomized controlled trials, and bench research studies that met the search criteria were all included for evaluation and data abstraction, while systematic reviews were excluded. Following article collection, an independent abstractor evaluated each publication for inclusion and manual data abstraction into a spreadsheet containing the following data points: virus/vector, viral family, study interventions based on the Centers for Disease Control and Prevention *Guideline for Disinfection and Sterilization in Healthcare Facilities (2008)*, other non-chemical interventions, affected party and reaction (if case report), duration of intervention, assessment of intervention, and study conclusions. Duplicate articles within the same viral family, case reports with no interventions, and publications on *in vivo* treatment options for clinical patients were removed. Two independent reviewers then validated the data points for accuracy and relevance to the primary research aim.

## Results

The comprehensive literature scoping review resulted in 4473 total publications and case reports related to the five designated viral-mediated vectors and associated disinfection terms (Table 2). Inclusion of taxonomic viral family and genus classifications in the search terminology resulted in publications related to clinical manifestations of the wild-type virus and *in vivo* treatment methods for patients. Therefore, 98.1% (n = 4390) of the total publications reviewed were deemed to be irrelevant to the primary research question of viral vector-mediated IPC methods. A subset of publications was further excluded (n = 59, 1.3%) due to the various alliterations of IPC methods utilized as search terms, which resulted in duplicate publications within the same viral vector category. The remaining 24 publications were represented within 20 unique journals, with 19 of the journals describing a peer-review process as part of manuscript submission.

**Table 2.** Comprehensive literature review results

Virus	Total Publications & case reports	Non-relevant publications and case reports removed	Relevant Publications and case reports	Duplicate Publications and case report removed	Total Relevant publications and case reports
**Adeno-associated virus**	841 (18.8%)	*830*	11	*8*	**3** (12.5%)
**Adenovirus**	975 (21.8%)	*959*	16	*8*	**8** (33.3%)
**Herpes simplex virus**	790 (17.7%)	*762*	28	*16*	**12** (50%)
**Lentivirus**	905 (20.2%)	*888*	17	*16*	**1** (4.2%)
**Retrovirus**	962 (21.5%)	*951*	11	*11*	**0** (0%)
**TOTAL**	4473	*4390*	83	*59*	**24**

Table 3 provides a comprehensive overview of IPC methods for viral vector-based gene therapy products. Chlorine compounds were found to inactivate AAV and AV, while alcohol-based disinfectants were ineffective. There was a relative paucity of studies investigating surface-based disinfection for HSV, however alcohol-based disinfectants were effective in one study. Ultraviolent (UV) irradiation was also found to inactivate HSV in numerous studies. No studies investigated disinfection for LV and retrovirus vectors.

**Table 3.** Infection prevention and control methods for common viruses utilized in viral-mediated gene therapy

Viral vector	Author, Year	Method of disinfection											Overview of findings
		*Alcohol*	*Chlorine compounds*	*Dry heat*	*Hydrogen Peroxide*	*Iodophors*	*Ortho-phthalaldehyde*	*Peracetic acid*	*Peracetic Acid & Hydrogen Peroxide*	*Phenolics*	*Quaternary ammonium*	*Ultraviolet radiation*	
Adeno-associated virus (AAV)	Howard, 2017^15^	X	✓	—	—	✓	—	✓	—	—	X	—	Autoclaving, 0.25% peracetic acid, iodine, and 10% bleach completely prevented AAV-mediated transgene expression.
	Tomono, 2019^16^	X	✓	—	—	—	—	—	—	—	—	✓	The activity of all rAAV serotypes was weakened by UV irradiation, NaOH, and NaClO exposure. Treatment for 10 days with tap water or 70% EtOH did not appreciably inactivate rAAV1, rAAV8, and rAAV9, but did affect the activity of rAAV2.
	Korte, 2021^17^	X	✓	—	X	—	—	✓	—	—	—	—	Sodium hypochlorite was the only substance resulting in complete and rapid (1 minute) capsid degradation, whereas potassium peroxymonosulfate required at least a 30-minute incubation time. PAA as a disinfectant should only be considered for the AAV2 serotype. Results reinforced previously published data to not use 70% ethanol or low concentrations of hydrogen peroxide for AAV disinfection.
Adenovirus (Adv)	Sauerbrei, 2009^18^ ^a^	—	—	✓	—	—	—	—	—	—	—	—	Viral inactivation of Adv serotype 5 required 2 hours of dry heat at 85°C.
	Tuladhar, 2012^19^	—	—	—	✓	—	—	—	—	—	—	—	HPV demonstrated complete inactivation by >4log_10_ reduction in Adv infectious particles on stainless steel.
	Moore, 2012^20^	—	—	—	—	—	—	—	—	—	—	✓	A UV-C exposure time of 6 minutes resulted in undetectable levels of Adv on both stainless steel and ceramic test surfaces.
	Romanowski, 2013^21^	—	—	—	—	—	—	—	—	—	—	—	A concentration of 50 PPM of PHMB was not virucidal against Adv at temperatures consistent with swimming pools or hot tubs.
	Goyal, 2014^22^	—	—	—	✓	—	—	—	—	—	—	—	HPV was virucidal (>4log_10_ reduction) to Adv at the lowest vaporized volume tested (25 mL).
	Gall, 2015^23^	—	✓	—	—	—	—	—	—	—	—	—	Demonstrated the dominant mechanism of Adv inactivation by chlorine is due to viral protein damage. Adv was inactivated at levels up to 99.99% by free chlorine still attached to host cells.
	Hoyle, 2016^24^	X	—	—	—	—	—	—	—	—	—	—	Adv serotype 4 was not eliminated by alcohol gel hand rubs alone and required soap and water handwashing during a healthcare outbreak in a pediatric ward.
	Ionidis, 2016^25^	X	—	—	—	—	—	—	—	—	—	—	The addition of 2.0% citric acid and 2.0% urea to the hand disinfectant was virucidal (>4log10) against Adv within 60 seconds. This formulation may be capable of inactivating all enveloped and non-enveloped viruses.
	Tsujimoto, 2010^26^	—	—	—	—	—	—	—	—	—	—	—	For 60 min incubation, 0.7 M arginine led to a sharp decrease in virus yield below pH 4.2 and had an undetectable virus yield at pH 3.8. For 5 min incubation, pH needed to be further decreased to result in same virus inactivation. Increasing temperature enhanced virus inactivation and enabled a higher pH to be used. Arginine-containing solvents synergize with pH and temperature for virus inactivation.
Herpes Simplex Virus (HSV)	Newcomb, 2012^27^	—	—	—	✓	—	—	—	—	—	—	—	Results demonstrated that 50 *m*M of hydrogen peroxide produced a model decrease in HSV titer (3–4 fold), but a lethal effect of 106-fold or greater was observed with the presence of sodium azide. Control data demonstrated sodium azide alone had little virucidal effect on HSV. The presence of catalase in HSV provides a significant level of protection against inactivation by hydrogen peroxide.
	Elikaei, 2013^28^	—	—	—	—	—	—	—	—	—	—	✓	A 1 μM concentration of MB with illumination for 45 minutes inactivated HSV (6.28 log reduction).
	Firquet, 2014^29^	—	—	✓	—	—	—	—	—	—	—	—	Full inactivation of HSV-1 was obtained in 7 seconds at 70°C and in 1 second at 100°C.
	Mirshafiee, 2015^30^	—	—	—	—	—	—	—	—	—	—	✓	A concentration of 50 *m*M of riboflavin with 1.29 *J/cm* ^2^ UV light resulted in inactivation of HSV (4.26 log_10_ reduction).
	Ren, 2016^31^	—	—	—	—	—	—	—	—	—	—	✓	UV-C light demonstrated a 99.96% inactivation of HSV particles with a mean exposure time of 10 seconds.
	Nardello-Rataj, 2016^32^	—	—	—	—	—	—	—	—	—	✓	—	Mixtures of these compounds (Aqueous solutions of didecyldimethylammonium chloride ([DiC10][Cl]) and octaethylene glycol monododecyl ether (C_12_E_8_)) demonstrated a wide spectrum of virucidal activity against lipid-containing deoxyribonucleic and ribonucleic acid viruses, including HSV. These mixtures could be used to extend the spectrum of virucidal activity commonly employed in numerous disinfectant solutions.
	Zakrewsky, 2016^33^	—	—	—	—	—	—	—	—	—	—	—	HSV was neutralized in the presence of dilute 1% CAGE.
	Elikaei, 2016^34^	—	—	—	—	—	—	—	—	—	—	✓	A concentration of 50 mM of riboflavin with UV light demonstrated a 4.44 log reduction of HSV in 10 minutes and a 6.09 log reduction in 15 minutes.
	van Kampen, 2017^35^	—	—	—	—	—	—	—	—	—	—	—	The infectious titers of 5.25 × 107 TCID50/mL of HSV-1 were reduced to below the limit of detection of the assay when samples containing 1% and 10% fetal bovine serum (FBS) were incubated with 0.1% SDS or 0.1% Triton X-100. Treatment with 0.1% Triton X-100 or 0.1% SDS reduced HSV-1 titers only by 1 log10 and 2 log10, respectively, when samples contained 98% FBS.
	Remy, 2018^36^	—	—	—	—	—	—	—	—	—	—	—	Triton X-100 0.1% is not sufficient to inactivate HSV-1 in high concentrations within serum. Triton X-100 1% led to complete inactivation of HSV-1 in 90% of serum samples. Complete inactivation of HSV-1 with 1% of Triton X-100 suggests that other enveloped viruses could also be completely inactivated by the same procedure.
	Dickinson, 2022^37^	✓	—	—	—	—	—	—	—	—	—	—	Testing with HSV virus demonstrated >99.99% efficacy in 60 seconds, consistent with broad-spectrum virucidal activity.
Lentivirus (LV)	Katoh, 2022^38^	—	—	—	—	—	—	—	—	—	—	—	Fabrics of level-3 surgical gowns serve better to reduce virus transmission compared to fabrics of chemical protective clothing with the same or higher barrier efficiency. Droplets of infectious body fluids may easily roll off fabrics with water-repellent finishing.
Retrovirus (RV)	N/A												

Abbreviations: AAV, Adeno-associated Virus; Adv, Adenovirus; CAGE, Choline and Geranate; EtOH, Ethyl Alcohol; HSV, Herpes Simplex Virus; HPV, Hydrogen Peroxide Vapor; LV, Lentivirus; MB, Methylene Blue; PAA, Peracetic Acid; PHMB, Polyhexamethylene Biguanide; Recombinant rAAV, adeno-associated virus; RV, Retrovirus; TCID, Tissue Culture Infectious Dose; UV, Ultraviolet; UV-C, Ultraviolet-C.

a No peer-review process is described within journal acceptance policies.

Key: ✓ partial or complete inactivation; X no inactivation; — not studied.

## Discussion

Novel gene therapies represent a significant breakthrough in the care of many medical conditions. Gene therapies utilizing viral vectors to deliver the genetic material present unique IPC considerations not present with traditional pharmaceutical formulations in the clinical setting, specifically concerning environmental disinfection and personal protective equipment. The NIH Recombinant DNA Guidelines, United States Pharmacopeia Chapters 800, and the sixth edition of Biosafety in Microbiological and Biomedical Laboratories (BMBL) offer the general framework of working with viral vectors, and they are often used as a reference for risk assessment for human gene transfer research.^9^ However, no single guidance document has comprehensive information about disinfection practices, shedding, and risk assessment when using these vector systems in a healthcare setting. Additionally, limited data on shedding requires organizations to work with their Institutional Biosafety Committee (IBC) and healthcare epidemiology teams to develop policies specific to their centers.^3,4^ In the last few years, several local IBCs have been requiring study teams to collect shedding data during the early phase of clinical trials. Hopefully, as there is growth in the field of human gene transfer in the coming few years more information related to shedding will be available. In general, most healthcare facilities recommend universal/standard precautions with patient material between 14 and 30 days after administration for both healthcare staff and direct family members.^9^

A hierarchy originally designed by Earle H Spaulding defines common disinfectants as either high-, intermediate-, or low level, based on their ability to kill various microorganisms. Disinfection protocols in the patient setting are extrapolated from this rational approach provided by Spaulding and from data generated from wild-type viruses in basic research studies. When placed on untreated plastic, recombinant AAV and adenoviral vectors were recoverable by cell culture for 3 and 14 days, respectively.^39^ Common oxidative disinfectants include peroxides, peroxygen-persulfate types, peroxide-peracetic acid, and chlorine-based disinfectants. These disinfectants have been found to successfully eliminate adenoviral vectors in research studies. These disinfectants have an appropriate spectrum of activity against some of the most common viruses in a research facility, given that any organism of equal or greater sensitivity than that of AVs likely also will be inactivated by these products.^40^

Similarly, the Environmental Protection Agency has a list of disinfectants for emerging viral pathogens that provides endorsement and kill claims based on active ingredient, virus type, and surfaces which are utilized for risk assessment during clinical trials by organizations.^41^ Based on this information, most therapies utilizing AV, AAV, or plasmid DNA vectors require disinfection with 1% sodium hypochlorite solution, with the need for prolonged contact times causing concern for damage to surfaces with repetitive long-term use.^4,41^ Similarly, center-wise policies related to disinfection are developed in consultation with local IBCs and infection prevention teams based on data gathered related to shedding. Herpesvirus can be inactivated with 70% alcohol solutions as well, presenting fewer material surface incompatibility concerns.^37^ Non-surface-based disinfectant options including hydrogen peroxide vapor and UV irradiation may also play a role in viral gene vector therapy disinfection protocols, however, effectiveness is dependent upon multiple factors including burden of organic matter which limits their usage as a primary disinfection agent.

Duration and extent of viral shedding varies with individual therapeutics, though data are limited.^1,3,4^ Standard precautions, including covering site of inoculation, should be utilized in patients treated with viral vector gene therapies. Additional transmission-based precautions with contact, droplet, and eye precautions should be employed when viral vectors are administered via aerosol. Immunocompromised healthcare workers or household contacts should avoid contact with patients treated with attenuated, replication-competent herpes viral vectors during the shedding period.^8,9,42^ When working with viral vector gene therapies, clinical staff should wear appropriate personal protective equipment, including gowns, gloves, and eye or respiratory protection, and should further be educated on the potential risks of percutaneous exposure through accidental needlestick.^7,9^

Commercially approved agents may provide healthcare teams with some product-specific disinfection and spill-related recommendations within their package inserts. Nadofaragene firadenovec, an AV-mediated therapy, recommends sodium hypocholorite with 0.5% active chlorine or 6% hydrogen preroxide solution with a contact time of 15 minutes to treat any local spill.^43^ Talimogene laherparepvec notes that any surface that comes in contact with the product should be treated with a virucidal agent, such as 1% sodium hypochlorite or 70% isopropyl alcohol.^42^ In the absence of clinical regulatory recommendations to support broad IPC methods for all commercial and clinical research therapies, institutions must develop local policies and procedures to cover a variety of operational scenarios.

Standardized, consistent, and easily interpreted recommendations must be established to ensure the safety of healthcare workers handling these products. Current handling recommendations for viral-based gene therapies should be derived from the United States Pharmacopeia Chapters 800, commercially available gene therapy package inserts, the Center for Disease Control and Prevention’s *Biosafety in Microbiological and Biomedical Laboratories (BMBL, 6* ^*th*^ *Edition)*, and the NIH Guidelines for Research Involving Recombinant or Synthetic Nucleic Acid Molecules.^9^ Table 4 provides institutional recommendations for infection control methods when handling or preparing viral-based gene therapies in a healthcare system.

**Table 4.** An infection prevention and control strategy based on single-center experience

Category	Recommendations
Pharmacy	
* Receipt & Storage*	Provide limited access to storage of products to ensure appropriate biosafety training prior to handling products.
* Compounding*	Biohazardous products should be prepared utilizing a primary engineering control, such as a Class II, Type A2, or greater biological safety cabinet, in order to provide an ISO 5 environment while compounding.
* Transport*	Transport in a leak-proof biohazard labeled closed container.
* Disinfection*	A 1:10 dilution of bleach can be utilized for complete germicidal activity within the pharmacy environment.
* Spill Control*	Spill kits should be readily available and contain all supplies, including a germicidal agent and PPE, to contain the spill. Large spills may require support from a trained hazardous waste professional.
* Disposal of PPE and Supplies*	PPE and disposable supplies are considered infectious waste and should be properly disposed of in containers clearly designated as biohazard.
Patient care	
* Administration*	Staff preparing and administering should wear personal protective equipment. Reduce aerosol production during preparation and administration procedures.
* Disposal of PPE and supplies*	PPE and disposable patient care items are considered infectious waste and should be properly disposed of in containers clearly designated as biohazard. Reusable patient care items and equipment should be cleaned and disinfected according to the manufacturer’s instructions for use.
* Disinfection & Cleaning*	A 1:10 dilution of bleach should be utilized for complete germicidal activity within the patient care environment.
* Spill Control*	To minimize the risk of exposure to personnel, PPE must be worn by the clean-up personnel. The type and volume of the waste spill will determine if gown, mask, goggles, etc. are needed. Minimally, personnel must wear gloves and follow blood and body fluid precautions. Handle any absorbent materials used to clean up the area as infectious waste and should be disposed of into a biohazard container. A spill kit may be used. Large spills may require support from environmental services.
* Post-Infusion Precautions*	In the inpatient setting, the patient should remain on Contact, Droplet, and Eye Precautions for the duration of hospitalization to account for potential dissemination of the virus/vector through secretions and/or excreta of the patient.
Employee health	
* Exclusion of Employees with High-risk immunocompromising conditions*	No reassignment is necessary if the appropriate post infusion precautions can be followed.
* Exposures*	Notify employee health immediately. Personnel who have had a recognized, unprotected exposure should monitor for symptoms of illness. Prophylaxis may be available for some vectors.

In this review, we highlight some of the challenges pharmacy and healthcare staff face regarding the use of virus-based gene therapies. The heterogeneity of methodology in studies included in this review precludes definitive recommendations of a single best infection control approach to these gene therapies, and further study will be needed as new products become available for patient use. Current advancements in gene therapy have opened the door to cures at a molecular level for many genetic diseases. The design of new experimental viral vectors with emerging technologies and the rate at which gene therapies are approved highlight the critical role of pharmacists, healthcare epidemiologists, infection preventionists, and biosafety professionals in identifying overall risk and operationalizing acceptable policies, predominantly in the absence of a consensus framework for the risk assessment process.

## Data Availability

The authors confirm that the data supporting the findings of this review are available within the article and supplementary material.
